# Perinatal Mood Disorders in Polish Women: A Cross-Sectional Study

**DOI:** 10.3390/jcm15052067

**Published:** 2026-03-09

**Authors:** Mariola Mróz, Agnieszka Marcewicz, Malwina Rezwow, Kamila Ciastek-Majtyka, Mateusz Cybulski, Grażyna Iwanowicz-Palus, Beata Pięta

**Affiliations:** 1 Department of Specialist Care in Obstetric, Faculty of Health Sciences, Medical University of Lublin, 4-6 Staszica St., 20-081 Lublin, Poland; agnieszka.marcewicz@umlub.pl (A.M.); grazyna.iwanowicz-palus@umlub.pl (G.I.-P.); 2 Holy Family Specialist Hospital SPZOZ, 25 Madalińskiego St., 02-544 Warsaw, Poland; malwaaa1234@gmail.com; 3 Midwife Femmed Midwifery Practice, Medical University, 50-425 Wroclaw, Poland; femmed@onet.pl; 4 Department of Integrated Medical Care, Faculty of Health Sciences, Medical University of Bialystok, 15-096 Bialystok, Poland; mateusz.cybulski@umb.edu.pl; 5 Department of Practical Midwifery Education, Poznan University of Medical Sciences, 60-355 Poznan, Poland; bpieta@ump.edu.pl

**Keywords:** mood disorders, quality of life, perinatal period

## Abstract

**Background**: Mood disorders such as postpartum blues, anxiety disorders, perinatal depression and posttraumatic stress disorder are common and can take various forms. For this reason, the assessment of emotional disorders and quality of life in women should be an integral part of health monitoring, and this was therefore adopted as the aim of the present study. **Methods**: The study was conducted using a diagnostic survey with questionnaires based on the authors’ survey, the SF-36, and the Patient Health Questionnaire-9. The cross-sectional study was conducted among 400 women in Poland, including 172 pregnant and 228 postpartum women. **Results**: Negative correlations were found between the PHQ-9 and SF-36 in pregnant and postpartum women. Selected obstetric factors were shown to significantly influence SF-36 and PHQ-9 scores (*p* < 0.05). **Conclusions**: The risk of mood disorders is associated with the duration of attempts to conceive, the course of pregnancy, and the number of hospitalizations. The presence of depressive symptoms affects the quality of life of women during the perinatal period.

## 1. Introduction

Pregnancy is a significant period in a woman’s life, marked by profound physical and psychological changes, as well as adjustments to her usual way of life [1,2,3].

Due to the ongoing changes, the quality of life during the perinatal period is becoming particularly important. Quality of life (QoL) is a complex concept that encompasses a subjective assessment of an individual’s physical, mental, and social functioning in relation to their goals, expectations, and lifestyle [4,5,6]. For many women, pregnancy, childbirth, and the postpartum period are times of positive changes and emotions; however, for some, it can become a source of significant stress, anxiety, and low mood [1,2,3].

Postpartum depression is a temporary condition characterized by mood swings, emotional hypersensitivity, and anxiety, and usually resolves spontaneously. Perinatal depression manifests as a prolonged low mood, loss of energy, and a sense of helplessness, which impedes a woman’s daily functioning and the care of her baby. It requires professional support. If left untreated, it negatively impacts a woman’s health and the child’s development. Postpartum traumatic stress disorder (PTSD) results from the traumatic experience of childbirth. Symptoms include intrusive, persistent thoughts, hypervigilance, and insomnia. Without appropriate care, symptoms can persist and worsen. Perinatal anxiety disorders include tension, excessive anxiety, and a sense of threat, leading to mental exhaustion. The most serious disorder is postpartum psychosis, which can pose a threat to the life of both the woman and her baby. Delusions, hallucinations, and a loss of contact with reality can occur [2,3,4].

Research indicates that women’s quality of life during the perinatal period can suffer significantly as a result of physical discomfort, sleep disturbances, hormonal changes, and concerns about motherhood and their baby’s health. Mood disorders, especially depression and anxiety, have a particularly detrimental effect on quality of life. A reduced quality of life is not only a consequence of these disorders but can also contribute to their aggravation, creating a vicious circle of emotional distress. Both sociodemographic factors, chronic diseases and obstetric factors—such as pregnancy complications, number of hospitalisations, or emergency childbirth—are also associated with a lower quality of life, often through the exacerbation of depressive and anxiety symptoms [4,5,6].

Mood disorders in the perinatal period are common and can take various forms [4,7]; however, analyses that simultaneously consider the course of pregnancy, medical experiences, and factors related to family planning are still needed. Moreover, the existing research has mainly focused on the diagnosis and treatment of mood disorders (including the improvement of well-being) or on previous depressive episodes as a risk factor for relapse. However, fewer studies have examined the impact of psychological support received prior to pregnancy on quality of life during the perinatal period. A better understanding of these relationships is crucial for the early identification of mood disorders and for designing effective preventive and supportive interventions. In this context, the aim of the present study was to assess the relationship between depressive symptoms and quality of life in pregnant and postpartum women, thereby contributing practical implications to perinatal care and extending existing knowledge.

The causes of emotional disorders are attributed to rapid hormonal changes, but also to psychological, social, and physical factors. Lack of support from a partner or the environment, social isolation, previous experiences related to mental health issues, and cultural pressure to be “the perfect mother”—all these factors increase the risk of mood disorders and reduce quality of life. Furthermore, fear of stigmatisation may prevent women from seeking help, which delays diagnosis and the initiation of appropriate treatment [7,8,9,10,11,12].

Early identification of symptoms and prompt intervention are crucial to preventing the long-term effects of mood disorders, both for the mother’s mental health and the child’s development. Effective treatment should be comprehensive, including psychotherapy, possibly medication, and broadly understood social support. At the same time, it is advisable to monitor quality of life as an indicator of women’s well-being—both in clinical and social contexts—to significantly improve the effectiveness of perinatal care and promote better adjustment of new mothers to their new life role [7,13,14,15].

## 2. Materials and Methods

The cross-sectional study was conducted between March and May 2023 among women hospitalised in selected healthcare facilities in Poland during the perinatal period. The main objective of the study was to determine whether there is a relationship between mood disorders and the quality of life of women during the perinatal period. The specific objectives included the following assessment: do obstetric factors, such as the time spent trying to conceive, the normal course of pregnancy, and the number of hospitalizations, affect mood disorders and the level of quality of life in women during the perinatal period? Does the use of psychological support before pregnancy influence the occurrence of mood disorders and the level of quality of life in women during the perinatal period?

The sampling of respondents was purposive. Participants had to meet the study criteria. The inclusion criteria included the patient’s age of majority, voluntary informed consent to participate in the study, and perinatal status, i.e., pregnancy (second or third trimester) or postpartum period. Exclusion criteria included diagnosed mental illness of the mother, death of a newborn, severe illness of the child, and premature birth for ethical reasons. Patients with traumatic experiences were excluded for ethical reasons, as completing the questionnaire could have intensified difficult emotions, and the researchers were unable to provide them with adequate psychological support. Furthermore, the study excluded women outside the perinatal period, those who refused to complete the questionnaire, and those with incomplete questionnaires.

The study was reviewed and approved by the Bioethics Committee of the Medical University of Lublin (KE 0254/72/03/2023) and conducted in accordance with the Declaration of Helsinki.

Each participant received a questionnaire after a discussion of its purpose, procedure, and method of completion. Respondents were also informed that their participation in the study was voluntary and anonymous, and that the findings would be used solely for scientific purposes. After completing the questionnaire, respondents placed it in a sealed ballot box, which was opened after the data collection was completed.

A total of 415 women participated in the study, of whom 400 correctly completed questionnaires were returned, including 172 from pregnant women in the first (11–14 weeks of gestation) and third trimester (33–37 weeks of gestation), and 228 from women in the postpartum period. The return rate for all questionnaires eligible for statistical analysis was 96.38%.

The analysis of these two groups results from significant biological and psychosocial differences between pregnancy and the postpartum period. Pregnancy is a phase of anticipation and adaptation to the upcoming parental role, whereas the postpartum period involves direct experience of childcare, hormonal changes following childbirth, and the potential occurrence of specific difficulties such as postpartum depression or fatigue related to newborn care. These differences may influence the studied variables in different ways, which justifies analyzing them separately.

The study used a diagnostic survey with questionnaires. The following research instruments were used:

A questionnaire designed by the authors, developed for the purposes of the study, containing questions about respondents’ characteristics (socio-demographic variables) and the study topic.

Variables such as the use of psychological support, the normal course of pregnancy and the duration of trying to conceive a child were assessed based on women’s responses to a closed question included in the author’s questionnaire. The unit of measurement was years, and the data obtained were categorized for statistical analysis into the following categories: less than 6 months, 1 year, 2 years, and 3 years or longer. This variable was analyzed as a categorical variable, according to the adopted coding scheme.

The Medical Outcomes Study 36-Item Short-Form Health Survey assesses health-related quality of life. It consists of 11 questions, comprising 36 statements that measure eight domains: physical functioning, role limitations due to physical health problems, bodily pain, general health, vitality, social functioning, role limitations due to emotional problems, and mental health. The overall score is the aggregate of the eight individual subscale scores. The Cronbach’s alpha coefficient for individual subscales for the female group is 0.63–0.95 [16].

The Patient Health Questionnaire-9, PHQ-9, is designed for screening for depression. It consists of 9 questions that correspond to its specific symptoms. Each question is scored on a scale of 0 to 3 based on the frequency of the symptom over the last 2 weeks. A score of 3 indicates the most frequent occurrence of the symptom. The maximum score is 27 and indicates the highest possible severity of depression. The Cronbach’s alpha coefficient for the PHQ-9 is 0.7 [17].

### Statistical Analysis

The analyses were conducted using IBM SPSS Statistics 28.0. The collected data were analyzed using the SPSS Statistics software (version 28). Quantitative variables were described using the mean (M), median (Me), and standard deviation (SD). Qualitative variables were presented as frequencies (n) and percentages (%).

Normality of distributions was assessed based on skewness and kurtosis (±2). The *t*-test for independent samples (Welch’s correction) was used for between-group comparisons, and relationships were assessed using Spearman’s rho coefficient; Bonferroni correction was applied in multiple analyses. PHQ-9 and SF-36 predictors were analyzed using linear regression (including hierarchical models). Regression assumptions were met (VIF < 2; tolerance > 0.60; no outliers in the analysis of residuals). Significance was set at α = 0.05.

## 3. Results

Table 1 presents the characteristics of the respondents. Most women were aged over 30 (55%), had a tertiary education (61.1%), lived in a provincial capital (51.1%), were married or in a relationship (96.6%), were professionally active (67.8%), and reported good financial standing (50.5%).

Table 2 presents descriptive data for the variables studied. The differences between pregnant and postpartum women did not reach statistical significance in terms of mood disorders (*p* = 0.866) and quality of life (*p* = 0.108).

Negative correlations were found between PHQ-9 and SF-36 in pregnant and postpartum women (*p* < 0.004). The time spent trying to conceive was negatively correlated with PHQ-9 (*p* < 0.004) among pregnant women. The number of hospitalisations during pregnancy correlated positively with PHQ-9 and negatively with SF-36 in both groups (*p* < 0.001). The number of hospitalizations showed a strong negative correlation with quality of life and a moderate positive correlation with the severity of depressive symptoms in pregnant women, while in postpartum women, moderate correlations with both variables were observed (SF-35: −0.33, PHQ-9: 0.39), as shown in Table 3.

Table 4 shows the relationship between normal pregnancy and mood disorders and quality of life among pregnant and postpartum women. Women with normal pregnancies, compared to those with abnormal pregnancies, had lower PHQ-9 scores among pregnant women (M = 6.48 vs. 11.13; *p* < 0.001) and postpartum women (M = 7.08 vs. 9.82; *p* = 0.007), as well as higher SF-36 scores among pregnant women (M = 108.93 vs. 72.56; *p* < 0.001) and postpartum women (M = 106.95 vs. 81.00; *p* < 0.001).

Table 5 presents differences in the severity of depressive symptoms and quality of life depending on prior use of psychological support, with a division into pregnant and postpartum women. No statistically significant between-group differences were observed in any of the analyzed groups, either in terms of mood disturbances or quality of life (all *p* > 0.05).

Table 6 presents a multiple regression analysis explaining the relationship between obstetric factors and mood disorders among pregnant and postpartum women. Among pregnant women, the PHQ-9 score correlated negatively with time spent trying to conceive (*beta* = −0.22; *p* = 0.001) and with normal pregnancy (*beta* = −0.17; *p* = 0.048), and positively with the number of hospitalisations (*beta* = 0.46; *p* < 0.001). Among postpartum women, the PHQ-9 score correlated positively only with the number of hospitalisations (*beta* = 0.27; *p* < 0.001).

Table 7 presents a hierarchical multiple regression analysis explaining the relationships between obstetric factors, mood disorders, and quality of life among pregnant women. The introduction of the PHQ-9 score into the model significantly increased the explained variation in the quality of life of pregnant women (Model 2: *R*^2^ = 0.70; *p* < 0.001). The SF-36 score correlated positively and weakly with normal pregnancy (*beta* = 0.18; *p* = 0.002), negatively and weakly with the number of hospitalisations (*beta* = −0.23; *p* < 0.001), and negatively and strongly with PHQ-9 (*beta* = −0.58; *p* < 0.001).

Table 7 also presents a hierarchical multiple regression analysis explaining the relationships between obstetric factors, mood disorders, and quality of life among postpartum women. The introduction of the PHQ-9 score into the model significantly increased the explained variation in th quality of life of postpartum women (Model 2: *R*^2^ = 0.38; *p* < 0.001). The SF-36 score correlated positively with normal pregnancy (*beta* = 0.24; *p* = 0.002) and negatively with the PHQ-9 (*beta* = −0.47; *p* < 0.001), as shown in Table 7.

## 4. Discussion

Mood disorders in the perinatal period are a serious health problem, affecting both the mental health of the mother and the development of the child. Despite the available literature, many aspects of this issue remain unknown. Therefore, this research analysis aimed to better understand the scale of this phenomenon and to determine how mood disorders affect women’s quality of life.

The original research conducted among pregnant and postpartum women showed that quality of life is associated with levels of depression. Li et al. noted that depression is not only a mental disorder but also a factor that significantly reduces quality of life in a broader sense: it negatively affects physical and psychological well-being as well as social relationships. According to the authors of the cited study, interventions for this group of women should be holistic, including not only psychological support but also support for everyday functioning [4].

Slomian et al. pointed to the long-term consequences of reduced quality of life for mothers with mood disorders, where their mental health was reflected in the well-being of their children. It affected their emotional and social development [18]. Da Costa et al. observed a strong correlation between the severity of depressive symptoms and reduced quality of life, particularly in mental health, emotional functioning, and social relationships among postpartum women. Women with depression experienced difficulties in relationships and daily parenting [19]. Similarly, Papamarkou et al. emphasized the need for psychosocial support to mitigate these effects on close relationships [20].

The study observed a negative correlation between the duration of trying to conceive and the severity of depressive symptoms. However, no statistically significant association was found with the SF-36 score. In this area, it is worth noting the results reported by Celda-Belinchón et al.: a higher number of treatment cycles and a longer duration of infertility were strongly correlated with quality-of-life indicators. Women reported reduced life satisfaction, particularly in the emotional, physical, and social dimensions [21]. Analyses conducted by Hasan et al. also indicated a strong correlation between stress levels and socio-economic status. The high cost of infertility treatment procedures caused higher levels of stress and depression in women with lower economic status [22]. On the other hand, having children in the past has been identified as a protective factor in relationships with partners [21]. Ni et al. showed that the more support women received from their families and society, the better their quality of life was [23]. Furthermore, negative emotions also mediate between quality of life and infertility [24].

Available academic research has indicated that hospitalisation of pregnant women is a factor that directly limits interpersonal contact and the fulfilment of social and family roles. As a result, feelings such as loneliness, lack of efficacy, and loss of control were observed to intensify [25].

Our research has shown that more frequent hospitalisations may be associated with variations in mental health and overall well-being. Toscano et al. found that a significant proportion of hospitalised pregnant women showed symptoms of depression or anxiety, with rates higher than in the general pregnant population [26]. Sade et al. highlighted inadequate diagnostic programmes that hinder early identification of risk factors, while psychological or psychiatric treatment is not always implemented, perpetuating the problem [27]. Similar findings were reported by Byatt et al., where high-risk pregnant patients did not receive adequate treatment despite depressive symptoms [28]. Other authors suggest that hospitalisation should be considered a marker of risk for emotional disorders and an indication for further diagnosis [29].

It is worth noting the link between stress and depression in the context of neuroimmunological theory. Numerous pregnancy complications, comorbidities, and pregnancy-induced illnesses can directly contribute to a deterioration in mental health and quality of life [15,30,31].

In the course of our research, we found that pregnant and postpartum women whose pregnancies progressed normally exhibited fewer symptoms of depression and a higher quality of life compared to women with abnormal pregnancies.

Ni et al. identified perinatal mood disorders as common. The risk of premature birth was a strong predictor of depressive disorders in the early postpartum period [23].

Kettunen et al. identified higher rates of depression among women who had previously been diagnosed with mental health problems. Furthermore, according to the authors, women with comorbidities and persistent vomiting during pregnancy were more prone to mood disorders and recurrent depression [32]. Our analysis did not show a significant association between the use of psychological help and mood disorders or quality of life in any of the groups.

The findings of Albertini et al., who compared the prevalence of depression in women with low and high obstetric risk, suggest that lower rates of depression occur in women with limited obstetric risk. The authors note that pregnancy complications may not be the dominant risk factors. A difficult financial situation and not having a partner are associated with a higher likelihood of depression [33]. Mothers who reported a strong attachment to their fetus showed less severe depressive symptoms. In contrast, those who reported dissatisfaction with interpersonal relationships had significantly more severe emotional disorders [19].

Rabinowitz et al. observed that the stage of pregnancy was associated with varying levels of mood disorders. These were most severe in the first trimester of pregnancy, which would explain the presence of somatic symptoms [34]. Boutib et al. reached similar conclusions, additionally noting correlations between quality of life and the well-being of pregnant women across particular trimesters [35]. The findings of Hu et al. demonstrated that the course of the third trimester, in particular, may determine a woman’s quality of life. It has been shown that focusing on the positive aspects of pregnancy can improve mothers’ quality of life [36].

As the analysed studies suggest, a pregnancy without complications is statistically associated with a lower severity of depressive symptoms. However, the absence of complications during pregnancy does not guarantee the absence of depression and does not justify abandoning screening programmes [37].

The study provided data on the relationships among mood disorders, obstetric factors, and women’s quality of life during the perinatal period. This highlights the need for systematic monitoring of patients’ mental health and physical condition by medical staff, who play a key role in the early diagnosis of worrying symptoms and referral to appropriate forms of support. At the same time, further in-depth research is needed to better understand the complexity of these relationships and to develop effective intervention strategies to improve the quality of life for women during the perinatal period.

### Strengths and Limitations of This Study

Our study used a standardized instrument, allowing other researchers to compare results and monitor changes. The strengths of this study also include personal communication with each respondent.

A strength of this study was the use of stratified analysis by perinatal status (pregnant and postpartum women). Many analyses consider only one of the groups. Our study allowed for a more precise analysis of the relationships in two physiologically and psychosocially distinct groups, which increases the substantive value of the study.

As for the limitations, it should be pointed out that this was a cross-sectional study, therefore causal relationships could not. Moreover, it was a national study conducted in Poland. Due to the use of purposive sampling and the analysis of a specific, non-representative group of participants, the obtained results refer only to the study sample and cannot be generalized to the broader population. Future studies should consider expanding the sample size and geographic.

## 5. Conclusions

The poorer quality of life of pregnant and postpartum women is associated with increased symptoms of depression and frequent hospitalizations.

A normal pregnancy and a short time spent trying to conceive are associated with lower rates of mood disorders.

Our results indicate the need for early diagnosis of mood disorders from the beginning of pregnancy. Preventive programmes should focus not only on diagnosis and treatment, but also on improving the psychosocial quality of life.

## Figures and Tables

**Table 1:** Participants’ characteristics.

Participants’ Characteristics		n	%
Age	<30	180	45
	≥30	220	55
Education	Primary education	39	6.4
	Secondary education	198	32.5
	Tertiary education	373	61.1
Residence	Urban—provincial capital	206	51.5
	Urban—other	118	29.5
	Rural	76	19
Relationship status	Married/in a relationship	386	96.6
	Single	14	3.4
Professionally active	Yes	271	67.8
	No	129	32.2
Self-reported financial standing	Very good	129	32.3
	Good	202	50.5
	Moderate	67	16.8
	Bad	2	0.4

**Table 2:** Descriptive statistics of mood disorders and quality of life in pregnant and postpartum women.

Variables		Min.	Max.	M	SD	*t* (*Welch*)	*p*	*d*
PHQ-9	Pregnant women	0.00	27.00	8.19	5.12	1.19	0.866	0.12
	Postpartum women	0.00	24.00	7.61	4.44			
SF-36	Pregnant women	27.00	150.00	95.81	30.13	−2.12	0.108	−0.22
	Postpartum women	36.00	149.00	101.94	26.53			

SF-36—The Medical Outcomes Study 36-Items Short-Form Health Survey, PHQ-9—Patient Health Questionnaire-9, *t*—test *t* Welch; *d*—effect size (Cohen’s d); *p*—values after Bonferroni correction.

**Table 3:** Correlation of Mood, Quality of Life, and Selected Obstetric Factors among pregnant and postpartum women.

Variables		SF-36	PHQ-9	Time Spent Trying to Conceive	Number of Hospitalisations
PHQ-9	Pregnant women	−0.78 ***	-	-	-
	Postpartum women	−0.56 ***			
Time spent trying to conceive	Pregnant women	0.17	−0.23 ***	-	-
	Postpartum women	−0.07	−0.01		
Number of hospitalisations	Pregnant women	−0.62 ***	0.48 ***	0.03	-
	Postpartum women	−0.33 ***	0.39 ***	0.02	

SF-36—The Medical Outcomes Study 36-Items Short-Form Health Survey, PHQ-9—Patient Health Questionnaire-9, *** *p* < 0.004 (Bonferroni correction).

**Table 4:** Relationships between normal pregnancy, mood disorders, and quality of life among pregnant and postpartum women.

Variables		Normal Course of Pregnancy				*t* (*Welch*)	*p*	*d*
		No		Yes				
		M	SD	M	SD			
PHQ-9	Pregnant women	11.13	5.87	6.48	3.71	5.74	<0.001	1.03
	Postpartum women	9.82	5.16	7.08	4.09	3.29	0.007	0.64
SF-36	Pregnant women	72.56	26.23	108.93	23.76	−9.08	<0.001	1.48
	Postpartum women	81.00	26.07	106.95	24.14	−6.02	<0.001	1.06

SF-36—The Medical Outcomes Study 36-Items Short-Form Health Survey, PHQ-9—Patient Health Questionnaire-9, M—mean, SD—standard deviation, *t*—test *t* Welch; *d*–size effect (Cohen’s d); *p*—values after Bonferroni correction.

**Table 5:** Analysis of the relationships between past use of psychological assistance, mood disorders, and quality of life among pregnant and postpartum women.

Variables		Past Psychological Assistance				*t* (*Welch*)	*p*	*d*
		No		Yes				
		M	SD	M	SD			
PHQ-9	Pregnant women	7.94	5.63	8.66	3.97	−0.98	1.000	0.14
	Postpartum women	7.17	4.54	8.41	4.16	−2.08	0.154	0.28
SF-36	Pregnant women	98.94	30.48	89.83	28.77	1.93	0.223	0.30
	Postpartum women	105.01	25.35	96.26	27.87	2.33	0.084	0.33

SF-36—The Medical Outcomes Study 36-Items Short-Form Health Survey, PHQ-9—Patient Health Questionnaire-9, M—mean, SD—standard deviation; *t*—test *t* Welch; *d*—size effect (Cohen’s d).

**Table 6:** Multiple regression analysis explaining the relationship between obstetric factors and mood disorders among pregnant and postpartum women.

	Predictors	B	SE	*Beta*	*t*	*p*
Pregnant women	(Constant)	5.71	1.37		4.17	<0.001
	Time spent trying to conceive	−0.93	0.27	−0.22	−3.48	0.001
	Normal pregnancy	−1.76	0.88	−0.17	−1.99	0.048
	Number of hospitalisations during pregnancy	2.51	0.46	0.46	5.47	<0.001
	Past psychological assistance	−0.19	0.68	−0.02	−0.27	0.786
*R*^2^ = 0.35; *F*(4.166) = 22.62; *p* < 0.001						
Postpartum women	(Constant)	5.94	1.29		4.59	<0.001
	Time spent trying to conceive	−0.12	0.26	−0.03	−0.47	0.641
	Normal pregnancy	−0.95	0.89	−0.08	−1.07	0.284
	Number of hospitalisations during pregnancy	1.39	0.40	0.27	3.44	<0.001
	Past psychological assistance	1.12	0.58	0.12	1.92	0.057
	*R*^2^ = 0.13; *F*(4.223) = 7.99; *p* < 0.001					

Dependent variable: mood disturbances. B—unstandardised regression coefficient; SE—standard error; *beta*—standardised regression coefficient; *t*—Student’s *t*-test statistic; *F*—*ANOVA* statistic; *R*^2^—adjusted coefficient of determination; *R*^2^—change in *R*^2^.

**Table 7:** Regression Analysis of Obstetric Factors, Mood Disorders, and Quality of Life in Pregnant and Postpartum Women.

		Predictors	B	SE	*Beta*	*t*	*p*
	Model 1	(Constant)	110.73	7.15		15.48	<0.001
Pregnant women		Time spent trying to conceive	3.94	1.39	0.16	2.84	0.005
		Normal pregnancy	17.08	4.61	0.27	3.70	<0.001
		Number of hospitalisations during pregnancy	−15.88	2.40	−0.49	−6.62	<0.001
		Psychological assistance	−1.94	3.57	−0.03	−0.54	0.587
	*R*^2^ = 0.48; *F* (4.166) = 40.83; *p* < 0.001						
	Model 2	(Constant)	130.19	5.72		22.78	<0.001
		Time spent trying to conceive	0.79	1.09	0.03	0.72	0.473
		Normal pregnancy	11.09	3.55	0.18	3.13	0.002
		Number of hospitalisations during pregnancy	−7.33	1.98	−0.23	−3.70	<0.001
		Psychological assistance	−2.57	2.71	−0.04	−0.95	0.344
		PHQ-9	−3.41	0.31	−0.58	−11.06	<0.001
	*R*^2^ = 0.70; Δ*R*^2^ = 0.22; *F*(5.165) = 80.97; *p* < 0.001						
		**Predictors**	**B**	**SE**	***Beta***	***t***	***p***
Postpartum women	Model 1	(Constant)	98.86	7.42		13.33	<0.001
		Time spent trying to conceive	−1.07	1.49	−0.04	−0.72	0.470
		Normal pregnancy	18.98	5.08	0.28	3.74	<0.001
		Number of hospitalisations during pregnancy	−5.12	2.32	−0.17	−2.21	0.028
		Psychological assistance	−8.28	3.35	−0.15	−2.47	0.014
	*R*^2^ = 0.18; *F* (4.223) = 13.52; *p* < 0.001						
	Model 2	(Constant)	115.71	6.76		17.12	<0.001
		Time spent trying to conceive	−1.42	1.29	−0.06	−1.10	0.274
		Normal pregnancy	16.28	4.44	0.24	3.67	<0.001
		Number of hospitalisations during pregnancy	−1.18	2.07	−0.04	−0.57	0.571
		Psychological assistance	−5.11	2.94	−0.09	−1.74	0.084
		PHQ-9	−2.84	0.33	−0.47	−8.49	<0.001
	*R*^2^ = 0.38; Δ*R*^2^ = 0.20; *F* (5.222) = 28.69; *p* < 0.001						

Dependent variable: SF-36 in the group of pregnant women; dependent variable: SF-36 among postpartum women. B—unstandardised regression coefficient; SE—standard error; *beta*—standardised regression coefficient; *t*—Student’s *t*-test statistic; *F*—*ANOVA* statistic; *R*^2^—adjusted coefficient of determination; *R*^2^—change in *R*^2^.

## Data Availability

The raw data supporting the conclusions of this article will be made available by the authors on request.

## Abbreviations

QoL	Quality of Life
SF-36	The Medical Outcomes Study 36-Items Short-Form Health Survey
PHQ-9	Patient Health Questionnaire-9
